# A national population-based study provides insight in the origin of malignancies metastatic to the ovary

**DOI:** 10.1007/s00428-015-1771-2

**Published:** 2015-04-19

**Authors:** Jolien Bruls, Michiel Simons, Lucy I. Overbeek, Johan Bulten, Leon F. Massuger, Iris D. Nagtegaal

**Affiliations:** Department of Pathology 824, Radboud university medical center, PO Box 9101, 6500 HB Nijmegen, The Netherlands; PALGA Foundation, Utrecht, The Netherlands; Department of Obstetrics and Gynecology, Radboud university medical center, Nijmegen, The Netherlands

**Keywords:** Ovarian cancer, Mucinous ovarian carcinoma, Metastasis, Epidemiology

## Abstract

A significant proportion of ovarian malignancies consists of metastatic tumors, with a wide variety in site of origin. Differentiating between a primary and metastatic malignancy of the ovaries can be difficult and misdiagnosis might have considerable impact on both treatment and prognosis. To further examine the origin of malignancies metastatic to the ovary, we performed a large-scale, nationwide search for ovarian metastases in the Dutch Pathology Registry (PALGA). All pathology reports concerning malignancies metastatic to the ovary and associated primary tumors in the Netherlands between 2000 and 2010 were collected. Age, year of diagnosis, tumor type, location of the primary tumor, and side of the ovarian tumor were extracted from the database. We identified 2312 patients fulfilling our selection criteria. The most common primary malignancy sites were colon (33.2 %), endometrium (17.1 %), breast (14.3 %), appendix (7.3 %), and stomach (4.5 %). The metastases were most frequently bilateral (46.3 %) followed by unilateral metastases in the right (26.7 %) and left ovary (19.8 %), while side was unknown in 7.2 % of cases. Of colorectal carcinomas, only 40.2 % metastasized bilaterally, compared to 63.9 % of breast, 62.9 % of gastric, and 58.9 % of appendix carcinomas. Left-sided colorectal carcinomas most often metastasized to the left ovary (*p* < 0.0001). We found colon carcinomas to be most frequently responsible for metastases to the ovaries, followed by endometrial and breast carcinomas. Metastases from breast, stomach, and appendix carcinomas were mostly bilateral, whereas metastases from colorectal carcinomas were mostly unilateral. The mechanisms underlying preferred sites for metastasis or side remain unclear.

## Introduction

Malignancies metastatic to the ovary are estimated to account for 5–30 % of all ovarian malignancies [1–8]. These most commonly originate from the colorectum, followed by endometrium, stomach, appendix, and breast [1, 2, 6–9]. Differentiating between a primary and metastatic malignancy of the ovaries can be complex as many metastatic carcinomas mimic primary ovarian carcinomas [9]. Mucinous ovarian carcinomas (MOC) are notoriously difficult to distinguish from metastatic adenocarcinomas, and as a consequence, the latter are often misdiagnosed as primary tumors [10–13]. A small study [11] claimed that as many as 40 out of the 52 MOC turned out to be metastases. However, the exact incidence of metastatic disease within the group of MOC is hard to determine. Algorithms as suggested in previous studies [8, 11] are not sufficient to identify all carcinomas metastatic to the ovary. Overlapping marker patterns between gynecological and gastrointestinal malignancies limit the usefulness of immunohistochemistry [9].

Since the treatment of choice of primary ovarian malignancies is different from that of metastatic disease to the ovary, misdiagnoses may have important consequences. As survival data are mostly based upon series containing a mixture of metastatic carcinomas and MOC, these are most likely biased. Although several studies focused on malignancies metastatic to the ovary, most were based upon small patient populations. Therefore, which malignancies most often metastasize to the ovary and which underlying pathways might be involved remains largely unclear. The purpose of the present large-scale, nationwide study was to gain more insight in primary tumor sites.

## Materials and methods

### Study population

**Fig. 1:**
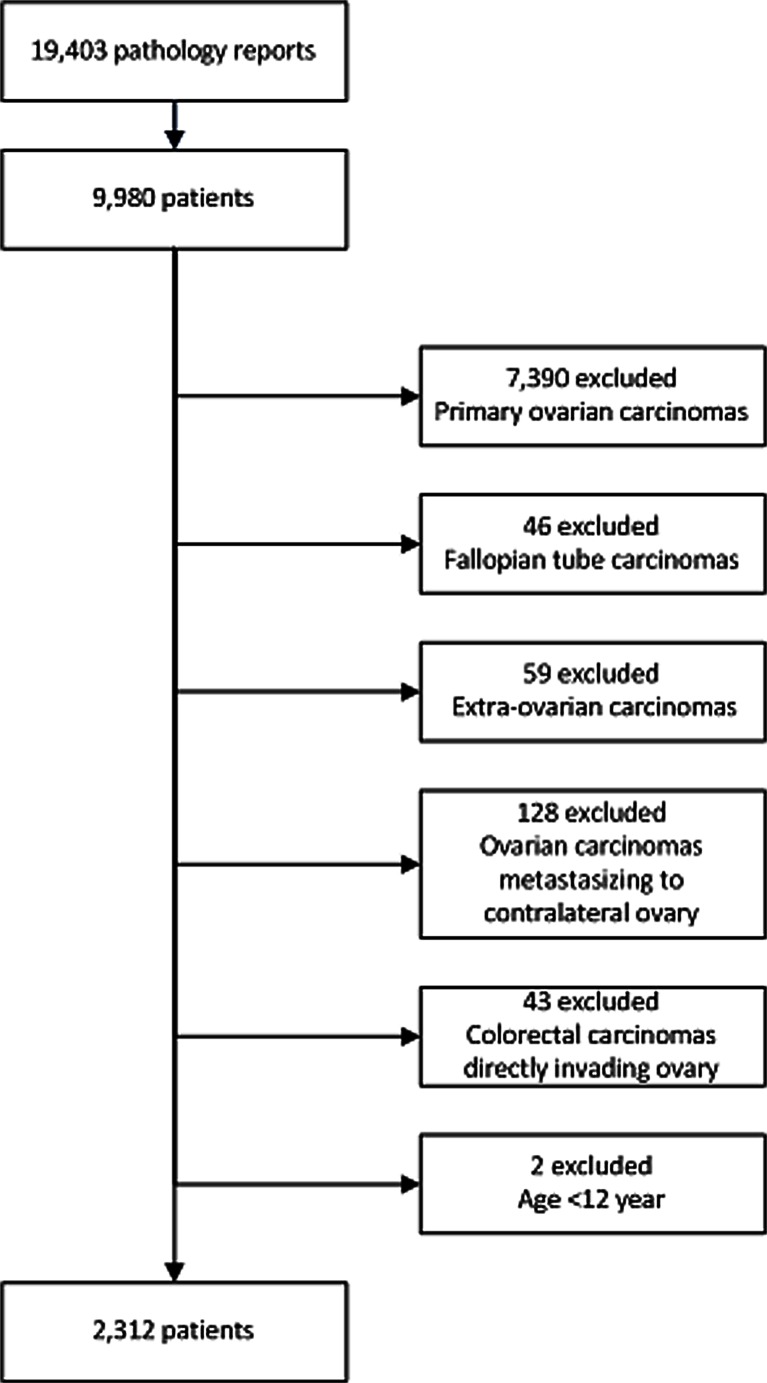
Flow chart of exclusion process

The nationwide network and registry of histo- and cytopathology in the Netherlands (PALGA) codes and saves pathology reports in the Netherlands as of 1971 with nationwide coverage as of 1991 [14]. The search terms “ovary” and “all metastases” were used for the years 2000–2010 to collect all pathology reports concerning malignancies metastatic to the ovary. Conclusions of reports were used for further data analysis. A flow chart of the exclusion process is depicted in Fig. 1.

For each patient, the following items were collected from the pathology report: age at time of diagnosis of ovarian metastasis, year of histological diagnosis, histological type and location of the primary malignancy, and side of the involved ovary. Survival data were not accessible.

Only metastases of which the primary site had been histologically verified were included, and these were classified per organ. Primary squamous or basal cell carcinomas of the skin were excluded. Cases with diagnoses based upon cytology only were excluded from the study. When a carcinoma was clearly not an ovarian primary but histological evidence identifying the primary site was lacking, it was classified as “primary site unknown.” Malignancies of which the distinction between primary and metastatic could not be made with certainty were labeled as “indeterminate site.” For endometrioid carcinomas, several histological criteria have been proposed to distinguish two synchronous primaries from one tumor being a metastasis from the other, although it is generally accepted that these are not always conclusive and definitive distinction is often not possible [15]. Primary fallopian tube carcinomas were excluded since new insight suggests that these are at least partly precursors of ovarian neoplasms [16]. Carcinomas in the ovary were classified by histological type. Metastatic carcinomas of gastrointestinal tract origin were classified as mucinous carcinoma, unless the pathology report stipulated a different specific histological type. When signet-ring cells were present, the carcinoma was classified as signet-ring cell carcinoma. Carcinomas with more than one histological subtype were classified as mixed. If a colorectal carcinoma was reported to invade the ovary directly, it was excluded since these are considered T4 rather than M1 carcinomas according to the latest TNM classification. Peritoneal lesions composed of abundant extracellular mucin, containing scant simple to focally proliferative mucinous epithelium with little cytologic atypia or mitotic activity, were considered as disseminated peritoneal adenomucinosis (DPAM). Peritoneal lesions composed of more abundant mucinous epithelium with the architectural and cytologic features of carcinoma were considered as peritoneal mucinous carcinomatosis (PMCA). Not all reports mentioned specific criteria or immunohistochemical staining used to reach a final diagnosis. The data used was anonymous. The scientific committee of PALGA approved this study.

### Statistical analysis

All analyses were performed using SPSS (Statistical Package for the social science; SPSS Inc, Chicago, Illinois) version 20.0. The chi-square test was used to compare multiple nominal variables. Distribution of median age was compared using a Mann-Whitney *U* test, and the sign test was used to compare two nominal variables. A *p* value ≤ 0.05 was considered statistically significant.

## Results

### Patient characteristics

Our search identified 19,403 pathology reports concerning 9980 patients, of which we included 2312 patients (Fig. 1). Median age of all patients with a malignancy metastatic to the ovary was 59 years (range 15–95). Median age of patients with a primary carcinoma of breast (49.5, *p* < 0.0001), lung (46, *p* < 0.0001), stomach (55, *p* = 0.002), and skin (46.5, *p* = 0.018) was significantly lower and that of patients with a primary carcinoma of bladder (67, *p* = 0.021) or endometrium (64, *p* < 0.0001) significantly higher compared to that of patients with a primary carcinoma from other sites. Further details are listed in Table 1.

**Table 1:** Primary tumor sites, age, and laterality

Location of primary tumor		Number	Percent	Median age, years (range)	Laterality ovarian tumor		
					Left (%)	Right (%)	Bilateral (%)
Upper gastrointestinal tract	Esophagus	8	0.3	66.5 (46–87)	2 (33.3)	2 (33.3)	2 (33.3)
	Stomach	103	4.5	55 (17–86)*	14 (14.4)	22 (22.7)	61 (62.9)
	Duodenum	3	0.1	61 (53–66)	0 (0)	0 (0)	2 (100)
	Pancreas	22	1.0	63.5 (35–77)	1 (4.8)	8 (38.1)	12 (57.1)
	Extrahepatic bile duct/gallbladder	14	0.6	63.5 (37–83)	3 (21.4)	1 (7.1)	10 (71.4)
	Total	**150**	**6.5**	**58** (**17**–**87**)	**20** (**14.3**)	**33** (**23.6**)	**87** (**62.1**)
Lower gastrointestinal tract	Small intestine	38	1.6	59 (34–83)	6 (16.7)	10 (27.8)	20 (55.6)
	Appendix	169	7.3	59 (31–86)	19 (11.7)	48 (29.4)	96 (58.9)
	Cecum	141	6.1	62 (26–91)	27 (20.6)	44 (33.6)	60 (45.8)
	Colon ascendens	122	5.3	62 (15–89)	28 (24.6)	41 (36.0)	45 (39.5)
	Colon transversum	35	1.5	58 (29–81)	2 (6.3)	11 (34.3)	19 (59.4)
	Colon descendens	58	2.5	62 (25–84)	15 (28.8)	16 (30.8)	21 (40.4)
	Rectosigmoid	357	15.4	59 (26–95)	110 (34.9)	98 (30.5)	109 (34.6)
	Colon multilocular	13	0.6	50 (32–80)*	2 (16.7)	1 (8.3)	9 (75.0)
	Colon^a^	41	1.8	58 (29–81)	4 (11.4)	9 (25.7)	22 (62.9)
	Total	**974**	**42.1**	**60** (**15**–**95**)	**213** (**23.9**)	**276** (**31.0**)	**401** (**45.1**)
Breast		**330**	**14.3**	**49.5** (**31**–**86**)*	**43** (**14.5**)	**64** (**21.5**)	**190** (**64.0**)
Urological tract	Urachus	1	0.0	44	0 (0)	0 (0)	1 (100)
	Kidney	8	0.3	62 (42–88)	2 (28.6)	3 (42.9)	2 (28.6)
	Bladder	15	0.6	67 (34–78)*	5 (33.3)	6 (40.0)	4 (26.7)
	Total	**24**	**0.9**	**66** (**34**–**88**)	**7** (**30.4**)	**9** (**39.1**)	**7** (**30.4**)
Genital tract	Vagina	1	0.0	48	1 (100)	0 (0)	0 (0)
	Cervix	26	1.1	53 (36–88)	10 (38.5)	8 (30.8)	8 (30.8)
	Endometrium	395	17.1	64 (32–89)*	81 (21.4)	115 (30.3)	183 (48.3)
	Total	**422**	**18.2**	**64** (**32**–**89**)	**92** (**22.7**)	**123** (**30.3**)	**191** (**47.0**)
Primary site unknown		348	15.1	61 (19–89)	64 (19.3)	89 (26.9)	178 (53.8)
Indeterminate primary site		34	1.5	60.5 (39–80)	8 (25.8)	12 (38.7)	11 (35.5)
Other	Lung	19	0.8	46 (35–64)*	8 (44.4)	7 (38.9)	3 (16.7)
	Skin	10	0.4	46.5 (25–76)*	2 (22.2)	5 (55.6)	2 (22.2)
	Thyroid	1	0.0	48	0 (0)	0 (0)	1 (100)
	Total	**30**	**1.2**	**46.5** (**25**–**76**)	**10** (**35.7**)	**12** (**42.9**)	**6** (**21.4**)
Total		**2312**	**100**	**59** (**15**–**95**)	**457** (**19.8** %)	**618** (**26.7** %)	**1071** (**46.3** %)

Laterality was unknown in 166 cases (7.2 %)

^a^Exact location of primary tumor in the colon not reported

*p < 0.05 compared with median age of all metastases to ovary tumor

### Primary tumor sites

**Fig. 2:**
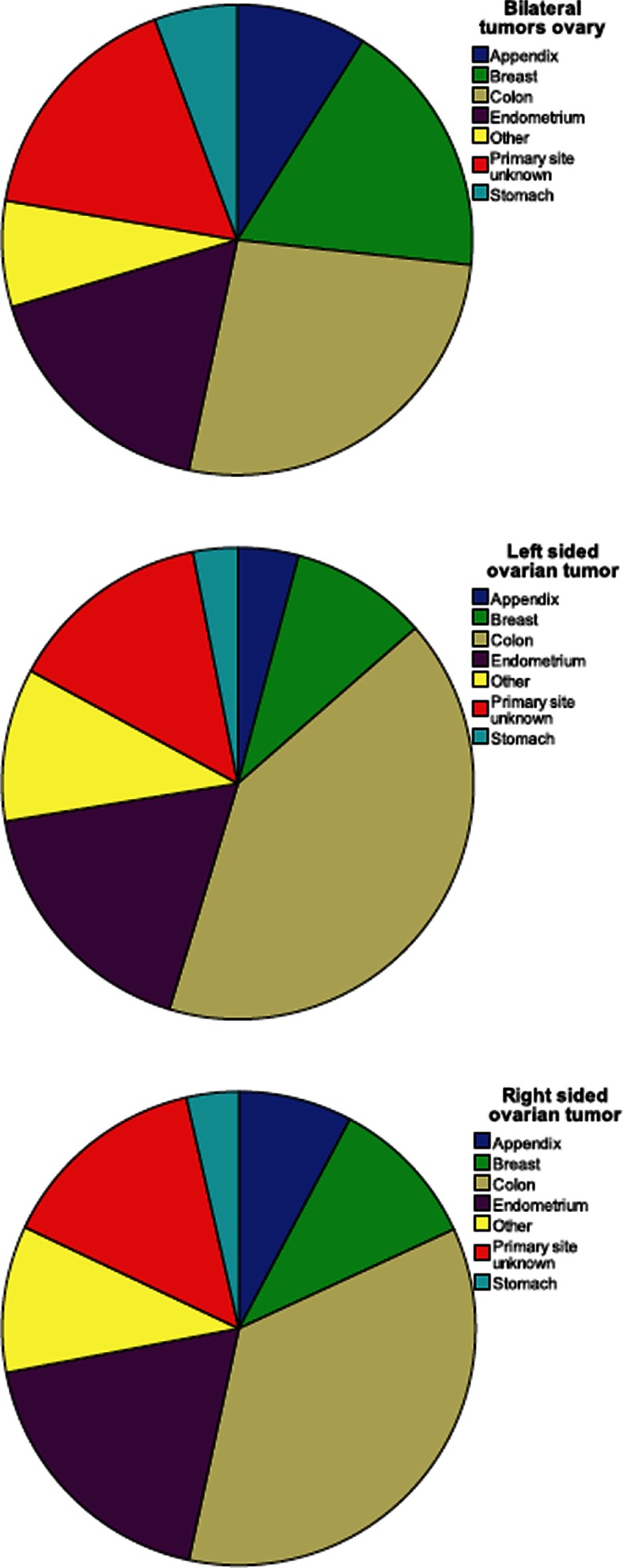
Side per site of origin

Of the 2312 malignancies metastatic to the ovary, the primary tumor was histologically confirmed in 1930 (83.4 %) cases. The most common primary site was colorectum (*n* = 767, 33.2 %), followed by endometrium (*n* = 395, 17.1 %), breast (*n* = 330, 14.3 %), appendix (*n* = 169, 7.3 %), and stomach (*n* = 103, 4.5 %), as depicted in Fig. 2.

In 348 cases (15.1 %), the primary site was unknown, but in 261 (75.0 %) of these, a primary location was suggested in the pathology report. Colorectum (*n* = 65, 18.7 %) was the most commonly suggested primary site, followed by stomach (*n* = 33, 9.5 %), upper gastrointestinal tract (gallbladder, pancreas or stomach) (*n* = 37, 10.7 %), breast (*n* = 26, 7.5 %), appendix (*n* = 25, 7.2 %) or other (*n* = 14, 4.1 %). Gastrointestinal tract without any specification of the location was suggested in 61 cases (17.5 %). In 87 cases (25.0 %), no primary site was suggested.

In 34 cases (1.5 %), the primary site was indeterminate. The remaining malignancies metastatic to the ovary (7.0 %) originated from rare primary sites. Further details on primary sites are listed in Table 1.

### Histopathology

Mucinous carcinoma was the most common histological type (*n* = 1082, 46.7 %), followed by signet-ring cell carcinoma (*n* = 182, 7.9 %), endometrioid carcinoma (*n* = 179, 7.7 %), and adenocarcinoma not otherwise specified (*n* = 108, 4.6 %). Mucinous carcinomas originated in 66.4 % (*n* = 717) from a colorectal carcinoma (CRC). For signet-ring cell carcinomas, the primary site was most often unknown (*n* = 60, 33.0 %), followed by stomach (*n* = 52, 28.6 %), colon (*n* = 34, 18.8 %), and appendix (16.5 %). Ductal and lobular carcinomas were responsible for respectively 7.1 % (*n* = 165) and 6.7 % (*n* = 156) of all metastases to the ovary (Table 2).

**Table 2:** Cases subdivided according to histology of ovarian tumor

Histological type	Number (%)
Adenocarcinoma	2124 (91.9)
Mucinous^a^	1167 (50.5)
Serous	76 (3.3)
Endometrioid	179 (7.7)
Clear cell	35 (1.5)
Signet-ring cell	182 (7.9)
Ductal	165 (7.1)
Lobular	156 (6.7)
Other^b^	164 (7.1)
Sarcoma^c^	68 (2.9)
Neuro-endocrine carcinoma	61 (2.6)
Squamous cell carcinoma	16 (0.7)
Transitional cell carcinoma	12 (0.5)
Melanoma	10 (0.4)
Unknown	21 (0.9)
Total	2312 (100.0 %)

^a^Includes 79 DPAM associated lesions

^b^Includes adenocarcinoma NOS, mixed, adenosquamous, undifferentiated, hepatoid, tubular, tubulopapillary, and villoglandular adenocarcinomas

^c^Includes 25 uterine sarcomas and 43 uterine malignant mesodermal mixed tumors

### Tumor side

Of all ovarian tumors, 46.3 % was bilateral, 19.8 % was left sided, and 26.7 % right sided. In 7.2 % of cases, side was unknown. The primary site of bilateral metastases was the colon in 26.6 %, breast in 17.7 %, endometrium in 17.1 %, appendix in 9.0 %, and stomach in 5.7 % of cases while 7.3 % were from a carcinoma in another organ and 16.6 % of unknown primary site. Distribution of primary tumor site was almost the same for metastases to the left and right ovaries as shown in Fig. 2.

**Fig. 3:**
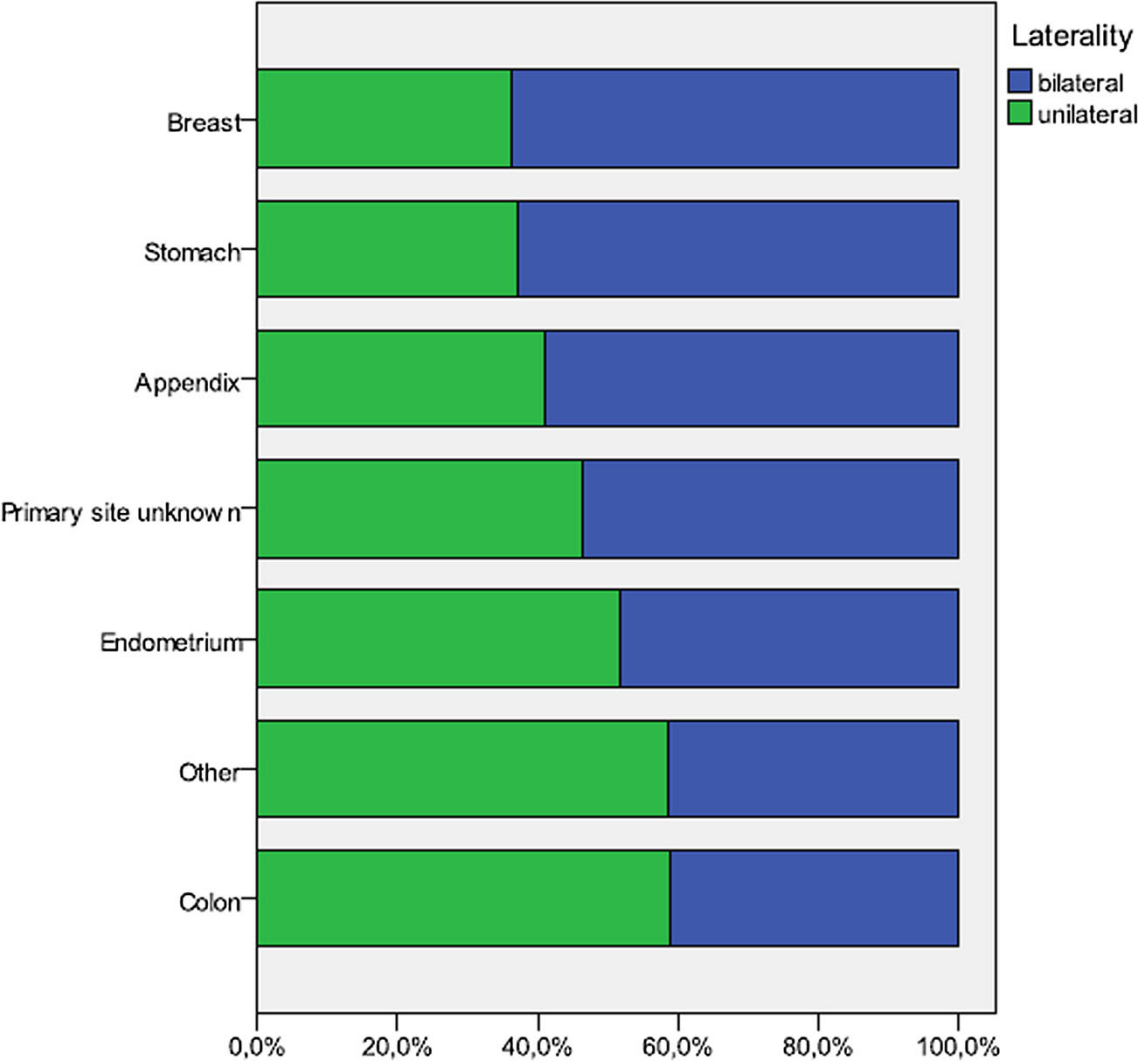
Uni- and bilaterality of ovarian metastases of most frequent sites of origin

Remarkably, only 40.2 % CRC gave rise to bilateral ovarian metastases, compared to 63.9 % of breast, 62.9 % of gastric, and 58.9 % of appendix carcinomas (Fig. 3).

Side of the primary CRC was related to which ovary was affected. Metastases to the left ovary originated more often from carcinomas in the left (descending colon and rectosigmoid) than from those in the right colon (cecum and ascending colon) (69.4 %, *p* < 0.0001). The difference was not statistically significant for metastases to the right ovary. Carcinomas of the appendix metastasized more often to the right ovary (71.6 %, *p* = 0.016). For no other organ was a difference in side found.

## Discussion

Differentiating between a primary ovarian malignancy and a malignancy metastatic to the ovary is challenging but important for prognosis and therapy. Our findings, on the as yet largest case series of 2312 primary malignancies metastatic to the ovary, are mostly consistent with earlier smaller studies with a majority of cases originating from gastrointestinal primaries (Table 3). Differences in distribution are partly associated with country of origin and partly due to small patient numbers. Endometrioid carcinomas are a particular problem as it might be difficult to distinguish between an ovarian metastasis of a primary in the endometrium and a synchronous primary endometrioid carcinoma in the ovary, as described earlier. Our dataset includes a relatively low number of uterine sarcomas and carcinosarcomas (less than 1 % of all cases) compared to some other studies. It must be noted that in all studies, the most frequent primary sites were consistently the gastrointestinal tract, breast, and endometrium [1–8].

**Table 3:** Studies examining frequencies of primary sites in metastases to the ovary

Author	Number	Large intestine (%)	Stomach (%)	Appendix (%)	Breast (%)	Endometrium (%)	Unknown origin (%)	Other (%)
Demopoulos 1987	96	12 (13)	6 (6)	1 (1)	32 (33)	14 (15)	0	31 (32)
Petru 1992^a^	82	23 (28)	22 (27)	0	28 (34)	0	0	9 (11)
Yada-Hashimoto 2003	64	7 (11)	15 (23)	1 (2)	9 (14)	15 (23)	1 (2)	16 (25)
Moore 2004^a^	59	19 (32)	4 (7)	12 (20)	5 (9)	0	10 (17)	9 (15)
Yemelyanova 2008^b^	142	46 (32)	5 (4)	26 (18)	0	0	0	65 (46)
De Waal 2009	116	23 (20)	7 (6)	2 (2)	32 (27)	23 (20)	9 (8)	20 (17)
Kondi-Pafiti 2011	97	15 (15)	24 (25)	3 (3)	15 (15)	22 (23)	0	18 (19)
Bruls 2013	2312	767 (33)	103 (5)	169 (7)	330 (14)	395 (17)	382 (17)	166 (7)

^a^Only non-genital primary tumor sites were included

^b^Only mucinous tumors were included

The relatively high incidence of metastases from CRC is remarkable. In the Netherlands, breast cancer is the most common type of cancer in women (31 %), whereas colorectal cancer accounts for 13 % [17]. Lung cancer is also fairly common (9 %) but relatively infrequently metastasizes to the ovaries. It is unclear why some primary tumor sites are more likely to metastasize to the ovaries than others.

Spread of appendiceal tumors to the ovaries is a notoriously complex phenomenon [18], which was also reflected in our findings. We found 65 low-grade appendiceal tumors (of which 62 associated with DPAM) and 104 appendiceal carcinomas (of which 24 associated with DPAM). Low-grade appendiceal tumor metastases in the ovaries were mostly also low grade, although some presented in the ovary as a carcinoma. Conversely, some appendiceal carcinomas manifested as DPAM, without ovarian involvement by carcinoma. PMCA was not diagnosed in any case which suggests that peritoneal involvement might have been underreported. However, extensive discussion of appendiceal tumors is beyond the scope of this study.

In our series, metastases from an unknown primary site made up the third largest group, with histological features suggestive of a primary site in 75 % of cases. Although this emphasizes the difficulties in distinguishing metastatic from primary malignancies, it is reasonable to believe that the actual number of cases with an unknown primary site is lower. For some patients, the primary site might have been diagnosed but considering factors such as age, physical condition, or prognosis, histological evidence might not have been sought. Incidentally, even though median age for several primary sites such as breast, lung, skin, endometrial, or bladder was significantly different, the wide range prevents age from being useful as a distinguishing feature.

The most frequently seen histological type of carcinoma was mucinous, originating from CRC in most cases. Primary ductal and lobular breast carcinomas were almost equally prevalent (7.1 and 6.7 % of our cases, respectively), even though lobular carcinomas account for only 5–15 % of all breast carcinomas. The loss of expression of the cell-cell adhesion molecule E-cadherin in lobular carcinomas has been suggested as an explanation for the more frequent spread of lobular carcinomas to the gynecological and gastrointestinal tract [19].

Side of the ovarian tumor (left or right) is a poor predictor of primary site, except for tumors originating from CRC. A left-sided ovarian tumor was more likely to originate from a left-sided CRC. Bilateral involvement of the ovaries occurred in half of the patients and was more common in patients with primary breast, stomach, and appendix carcinomas, whereas CRC more frequently presented with unilateral metastasis to the ovary. Interestingly, Petru et al. [20] found a significantly better 5-year survival rate in patients with unilateral compared to bilateral ovarian involvement. The different patterns of metastasis according to primary site, along with differences in survival, suggest that the metastatic routes followed might not be the same. Chang et al. [21] hypothesized that retrograde lymphatic spread might be responsible for ovarian metastases from gastrointestinal cancer. Our finding that most CRCs tend to metastasize only to the ipsilateral ovary suggests that continuous spread through the peritoneal cavity is involved. How often hematogenous spread is involved is not clear, although the tendency of breast cancer to metastasize to both ovaries supports this route. It is generally accepted that patterns of metastatic spread depend on specific characteristics of tumor cells with metastatic potential as well as the micro-environment of recipient tissue [22–24]. Between organs, properties of the extracellular matrix and vascular endothelial cells are different in terms of homing ligands and production of regulatory factors [25, 26]. While it is still not fully understood why some tumors preferentially metastasize to particular sites or bilateral rather than unilateral, direct transperitoneal spread is an important mechanism in our case series consisting mostly of primary malignancies in the abdomen or pelvis.

We only included histologically confirmed cases but did not revise histology. As for many cases, the criteria used to conclude that an ovarian malignancy was a metastasis from a primary elsewhere, were not available, these were not taken into consideration. While immunohistochemistry is often inconclusive, PAX-8 might prove useful as a marker relatively specific for mucinous ovarian carcinomas [27, 28]. A limitation of our study is its inclusion of patients from a geographically defined area only and in a specific time frame. However, comprehensive coverage of the whole Dutch population in the PALGA database does provide solid evidence of the site of primary malignancies metastatic to the ovary.

In summary, in our large patient series, colon is the most frequent primary site of a malignancy metastatic to the ovary, followed by endometrium and breast. Metastases from breast, stomach, and appendix carcinomas were mostly bilateral. Colorectal carcinoma metastases were mostly unilateral, with a trend for the side of CRC to correspond to the side of the ovarian metastasis. Mechanisms determining patterns of metastasis are still largely unclear, but our observations suggest that these might be different per primary tumor type.
